# Non-Destructive Evaluation of Thermal Aging in EPDM Rubber Using Electromagnetic Techniques

**DOI:** 10.3390/ma16155471

**Published:** 2023-08-04

**Authors:** Sobhan Sepehri, Stacy Trey, Kajsa Lake, Carl Cumming, Christer Johansson

**Affiliations:** 1 Digital Systems, RISE Research Institutes of Sweden, SE-411 33 Gothenburg, Sweden; 2 Materials and Production, RISE Research Institutes of Sweden, SE-164 40 Kista, Sweden; 3 2dFab AB, SE-853 50 Sundsvall, Sweden; 4 Trelleborg Mixing Forsheda AB, SE-331 71 Forsheda, Sweden

**Keywords:** EPDM, eddy-current sensing, electrical impedance measurements, non-destructive testing, sealants, hardness, graphene

## Abstract

This study investigates the use of eddy-current technology and impedance spectroscopy in sensing the change in rubber properties after it is exposed to accelerated thermal aging. The thermal aging process, by application of temperature and pressure over time, of ethylene propylene diene monomer (EPDM) rubbers containing both carbon black (CB) and graphene are investigated. Both eddy-current sensing and electrical impedance measurement techniques were used for electromagnetic analysis. Both methods measure the in- and out-of-phase responses as a function of excitation frequency at room temperature. The measurements were performed before and after the aging process. The electrical percolation threshold was detected in the rubber samples by varying the CB content from 0 to 40 wt%. In the rubber sample containing 30 wt% CB, 0–5 wt% of the CB was replaced with graphene flakes. The substitution of graphene for CB in the EPDM rubber formulation provided an enhanced eddy-current and electrical impedance response. The findings demonstrate the feasibility of employing electromagnetic analysis techniques to investigate the extent of aging.

## 1. Introduction 

Rubber sealants or gaskets are commonly used in a range of industries, including nuclear energy, oil and gas, and robotics, and they are often the weakest link in terms of aging and failure points [1]. They have polymer base compositions and come in various shapes depending on their application. The sealant material must be hard enough to withstand pressures, temperatures, and chemicals while being soft enough to conform to the two different surfaces that need to be kept tight from the leakage of gases and liquids. They are exposed to harsh environmental conditions such as high pressure, a large range of temperatures and humidities, along with cycling of these conditions, leading to sealant failure. To mitigate the risks associated with sealant failure, they must be inspected and replaced regularly. Sealants are usually less expensive and more disposable in comparison to other parts in a system, for example, in high-pressure processing systems composed of valves and pipes. In failure studies, it is often the sealants that are suspected of being incompatible with the environment or conditions. Sealant failure can lead to the loss of product material and cause environmental contamination, which could pose a serious risk to human health and safety. These sealants can be located in inaccessible areas, such as nuclear power reactors or buried underground. Therefore, replacing them can involve a stop in production, excavation, or other costly measures. Visual inspection and non-destructive methods are used to assess the condition of the sealants. However, the degradation and wear of these sealant materials is usually not easily measured. Therefore, they are usually replaced on a set schedule, regardless of their condition, or left until failure. Real-time monitoring of the sealant condition could allow for detecting the early signs of sealant material degradation, helping to prevent failure or unnecessary replacement. 

Numerous studies show that the thermal aging of rubber sealants affects their mechanical properties [2,3,4], and therefore it is of increasing interest for many industries to digitally connect sealants so that their condition can be determined without visual inspection. Demonstrations of this concept are present in academics but have not been achieved in practice. It has also been determined previously that thermal aging alters the electrical and dielectric properties of rubber sealants [5,6,7,8,9], and thus, by monitoring these properties, the aging of the rubber can be quantitively measured. 

Eddy-current sensing is a non-destructive, non-contact technique that could be used for monitoring the thermal aging of rubber. It is commonly used for the inspection and quality control of materials, especially metallic materials [10]. This method electrically inspects conductive materials using an alternating magnetic field to detect damage and cracks or measure coating thickness and conductivity. The eddy-current sensor response depends not only on the conductivity and magnetic permeability of the material but also on the permittivity of the material [11]. In eddy-current sensing, a time-varied magnetic field is created by applying an alternating current to a probing coil. Near a test sample, the field penetrates the material and generates continuous circular eddy currents. These induced currents generate a secondary magnetic field that opposes the primary field and weakens it. Monitoring the coil impedance variation in the presence and absence of the sample reveals information about the test material and its electrical conductivity, permeability, and permittivity. Eddy-current technology can thus be used to detect changes in the chemical and physical properties of rubber containing carbon black (CB), for example, in sealants. Here, this principle is used to monitor the change in electromagnetic properties of sealant material when a fraction of CB is replaced with graphene. The results indicate that replacing 0.5 wt% of CB with graphene in the rubber sealants improves not only the eddy-current signal but also the mechanical properties and resistance of the material to distortions arising due to rapid decompression or general aging.

Electrical impedance spectroscopy is another non-destructive method that could be used to monitor the aging of CB-filled rubbers. This analysis method measures both the capacitive (dielectric) and conductive (resistivity) properties of a sample over a specific frequency range [12]. By attaching electrodes and applying a time-varied electric field to the sample, the electrical properties of the rubber can be measured. 

Introducing nanomaterials into polymer matrices has proven to significantly influence polymer properties and performance [13,14]. Graphene is one of these nanomaterials that has attracted great scientific interest and is used to improve polymer properties. It is a two-dimensional, one-atom-thick honeycomb carbon atom structure with unique thermal, electrical, and mechanical properties. Well-dispersed graphene at low concentrations has been shown to improve the mechanical properties of the polymer by allowing the effective transfer of stress from the polymer matrix [15]. The high thermal and electrical conductivity of the added graphene, which is significantly higher than most conductive materials, enhances the thermal and electrical conductivity of the resulting polymer as well. This may amplify the electrical signal from the sealant when measured by eddy-current and/or electrical impedance equipment, providing greater differences in the signal after aging. There is also the possibility that the addition of a small amount of graphene can increase the permeability of the material [16]. In this work, we have substituted a small portion of CB filler in the rubber with graphene flakes to investigate the potential influence of graphene on the electrical properties of ethylene propylene diene monomer (EPDM) rubber.

The present study investigates if eddy-current technology and impedance spectroscopy can be used to sense the change in rubber properties after it is exposed to accelerated thermal aging. The CB concentration required for electrical percolation in this formulation of rubber is determined by measuring a range of CB from 10 to 40 wt%. A typical synthetic formulation for EPDM rubber commonly used for sealants was chosen for this purpose. A portion of the CB was substituted with graphene flakes (GP25), with an average particle size of 25 µm as a multifunctional nanomaterial. The eddy-current signal, impedance, and mechanical hardness with the addition of GP25 (0.5–5.5 wt%) before and after aging of the rubber samples are investigated.

## 2. Materials and Methods

### 2.1. Materials and Samples

Rubber samples were formulated and mixed at Trelleborg Forseda Mixing, Trelleborg, Sweden. The average sample size for the electrical measurements was a cylinder of approximately 13 mm in height and 30 mm in diameter. The EPDM formula was chosen to start with, as it is currently one of the most common synthetic rubber sealant materials used. The formulation comprised 100 parts per hundred rubber (phr), 70 phr CB (N550, with a typical average particle size of 40–70 nm and total surface area of 40 m^2^/g), 55 phr paraffin oil, 1 phr stearin, 1.5 phr activator, and 6 phr peroxide. The samples were volcanized at 175 °C for 15 min. The formulation used for the preparation of each EPDM rubber sample is given in Table 1.

In this formulation, CB was added in varying amounts from 10 to 40 wt% to determine the electrical percolation threshold. The electrical percolation threshold was found to be below 30 wt% CB. The formulation containing 30 wt% CB was chosen as a reference sample for electrical characterization, as it is above the percolation threshold and has a high electrical conductivity. Graphene flakes were substituted for three specific amounts of CB filling agent, and the effect on the resulting material eddy-current signal, electrical impedance spectroscopy, and mechanical hardness was investigated. Further, 2Dx^®^ graphene flake of a 25 µm flake size (average 100-layer size) from 2dFab AB, Sundsvall, Sweden, was used. The GP25 was added in three different concentrations (0.5%, 3.0 and 5.5 wt%) and substituted an equal amount of CB in the 30 wt% CB formulation. 

### 2.2. Eddy Current Setup

**Figure 1:**
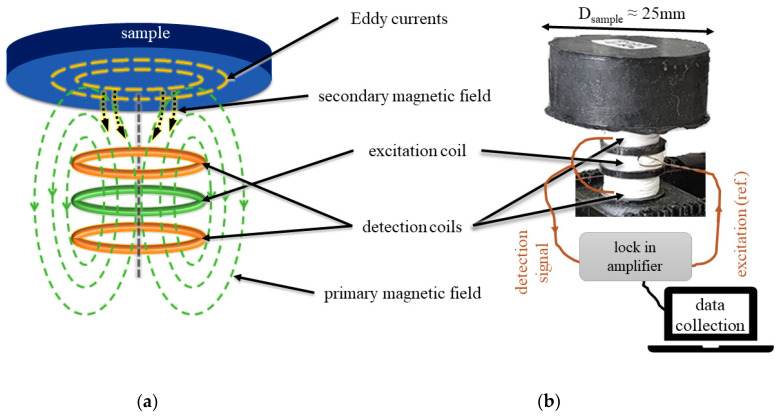
(**a**) Eddy-current differential coil system and (**b**) diagram of the performed measurement using the lock-in technique.

The eddy-current experimental setup consists of three individual coils: an excitation coil for generating the eddy current in the sample and two differentially connected pickup coils for receiving the secondary fields from the eddy current in the test sample. Figure 1a shows the coil system where the excitation coil is centered between the two detection coils, wound in opposing directions. This minimizes the induced voltages in the pickup coils from the excitation field. The pickup coils form a differential probe, having zero output voltage in the absence of a test sample. The samples are placed directly above the coil system, and the relative positions of the coil system and the sample are kept intact when changing the samples. The generated AC magnetic field amplitude from the measurement coil system at the center of the samples is in the range of 15 µT. The probing electric fields that are generated in the samples due to the applied AC magnetic field from the excitation coil are circular and perpendicular to the direction of the sample thickness.

To obtain accurate measurements of the small eddy-current signals, a phase-sensitive detection technique with a lock-in amplifier (SR-860, Stanford Research Systems, Sunnyvale, CA, USA) was used. In this method, the sample is exposed to an excitation field at a fixed frequency, and the sample response is measured by the pickup coils. The amplifier detects the response at the reference frequency and rejects all other frequency components using a phase-sensitive detector. The detector has a very narrow bandwidth filter, which removes noise sources close to the reference frequency and provides a high signal-to-noise ratio. A MATLAB program controls the instrument and collects the in-phase and out-of-phase components of the signal. The eddy-current sensor system is calibrated against paramagnetic Dy_2_O_3_ that only exhibits an in-phase response independent of the excitation frequency. Figure 1b shows a diagram of the described measurement. 

### 2.3. Electrical Impedance Analysis

The electrical impedance (amplitude and phase) versus excitation frequency was measured in the 20 Hz to 100 kHz range using an LCR HM8118 Rhode & Schwarz system. Cu electrodes were placed in good physical contact with the cylindrical samples in a plate capacitor-type configuration. The electrode area and sample thickness were measured to determine both the electrical conductivity and the in-phase permittivity using impedance amplitude and phase data. The permittivity of the sample was determined by comparing the calculated capacitance value to that of a vacuum (with permittivity 1) between the plate electrodes. The excitation voltage amplitude was set to 1 V, giving an electric field amplitude of about 77 V/m. Since the applied excitation voltage is between the two electrodes, the electric field is parallel to the thickness of the sample.

### 2.4. Mechanical Compression Set, Accelerated Aging, and Hardness Measurements

The samples were compressed (in the thickness direction) by 25% of their initial thickness according to ISO 815-1 [17], ASTM D395B [18], and placed in an oven at 125 °C. The samples were aged for 30 days. The thickness and diameter of the samples were measured before and after the aging process. They were measured 30 min after taking them out of the oven and removing the compression. The thickness that had not recovered after this time was recorded. The electrical impedance and the eddy-current measurements were also performed before and after the aging process. Hardness measurements were completed with an Elastocon micro Shore A system (hardness equipment, according to EN ISO 868 [19], ASTM D2240 [20]).

## 3. Results and Discussion

### 3.1. Eddy-Current Measurements

**Figure 2:**
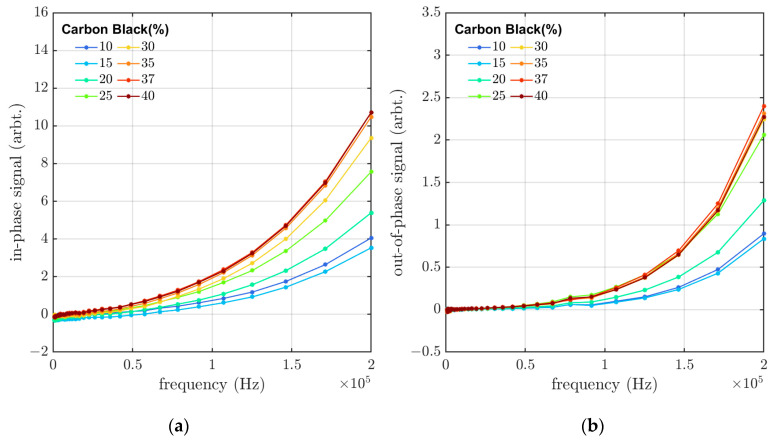
(**a**) In-phase and (**b**) out-of-phase eddy-current signals versus frequency for samples with CB concentrations ranging from 10 to 40 wt%, measured before the aging.

The rubber samples containing 10–40 wt% CB were measured with the eddy-current sensor system in the frequency range of 1–200 kHz. The results, taken from the samples before aging, can be seen in Figure 2. Increasing the concentration of CB lowers the resistivity of the material, leading to (as expected) higher eddy-current signal levels in both in-phase and out-of-phase components. This decrease in resistivity makes it easier for eddy currents to circulate in the material. The in-phase and out-of-phase signals for rubber samples with CB concentrations of 35 wt%, 37 wt%, and wt40% are very close to each other and almost overlap in the in-phase signal. Due to the percolation threshold of the samples, the increase in the CB concentration over 35 wt% does not affect the resistivity of the rubber material, and therefore the eddy-current response does not change with further addition of CB. However, there is a deviation in the results for the samples containing 15 wt% CB. With a CB concentration under the percolation threshold, it is expected that the in-phase component of the eddy-current response should be greater with increasing CB concentrations. The 15 wt% CB sample does not follow this trend and has lower conductivity in comparison to the 10 wt% CB sample. 

In Figure 2, we can see that both the in-phase and out-of-phase responses increase with higher frequencies. The induced voltage that creates the circulating eddy currents in the sample is proportional to the rate of change in the magnetic field with time ($\frac{d\phi }{dt}$, where *ϕ* is the magnetic flux and *t* is time). As the frequency of the magnetic field increases, the rate of change in the magnetic field also becomes greater, resulting in a stronger induced eddy current in the samples. In this eddy-current sensing method, it is possible to induce eddy currents, both in individual carbon black particles as well as in larger diameter eddy-current loops when the carbon black particle concentration is high enough for the particles to form a network (in physical contact with each other) above the percolation threshold.

**Figure 3:**
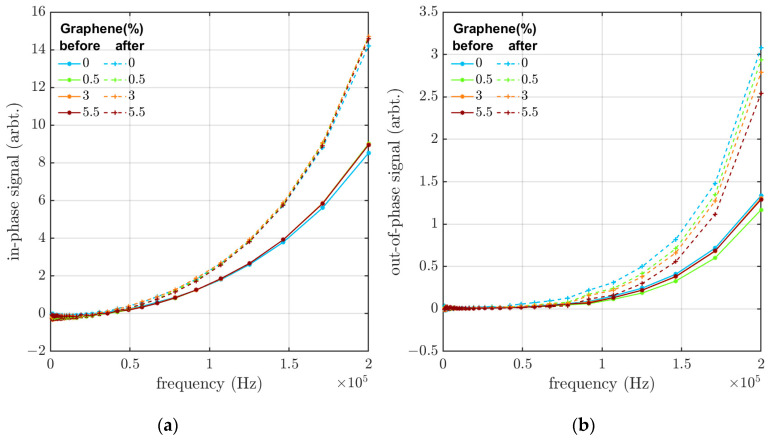
(**a**) In-phase and (**b**) out-of-phase eddy-current response versus frequency before and after aging using different graphene concentrations in the samples (with a CB concentration of 30 wt%).

The in-phase and out-of-phase components of the eddy-current signal were also measured for the 30 wt% CB samples containing 0 to 5.5 wt% of GP25. As observed in Figure 3, exchanging the CB with GP25 does not affect the eddy-current response considerably. This indicates that although the GP25 flakes have a higher aspect ratio and are, on average, larger in size (25 µm) compared to the CB particles (average 40–70 nm), there seems to be no significant change in the electrical percolation path in the samples. This could be a result of poorly dispersed graphene because of the mismatched solubility parameters. This could cause decreased interactions between the materials and lower adhesion between the GP25 flakes and the rubber matrix, affecting the final properties of the composite [21]. It is also possible that the GP25 flakes were crushed in the processing of the EPDM rubber samples, meaning that the full advantages of the increased aspect ratio cannot be realized or that the percolation level had already been achieved, which is why no large increase in signal was obtained by substituting CB with GP25.

Samples with 30 wt% CB and various concentrations of GP25 were aged, as described in Section 2.4. Figure 3 shows that, for all graphene concentrations, the eddy-current response increases with the thermal aging of the sample, indicating that the conductivity or dielectric response of the samples increases with thermal aging. The relative increase in the eddy-current response is slightly higher in samples with 0.5 wt% graphene (compared to the other graphene concentrations), even if the responses prior to aging were relatively similar.

### 3.2. Electrical Impedance Measurements

**Figure 4:**
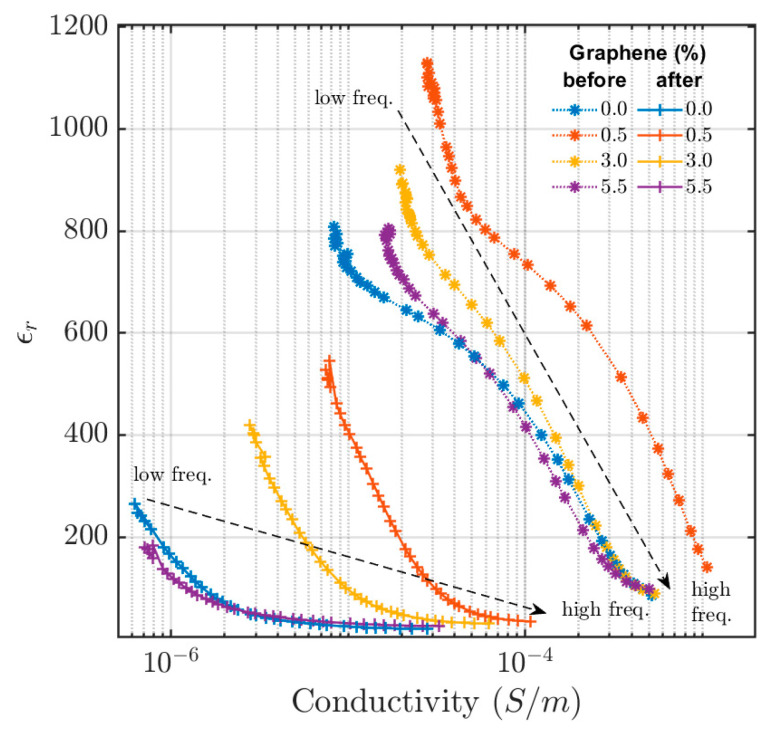
In-phase permittivity versus electrical conductivity for the sample with 30 wt% CB concentration at various graphene concentrations before and after the aging process. Data points are obtained at different excitation frequencies ranging from 20 Hz to 100 kHz (starting from the top at low frequency and large permittivity and falling in permittivity with increasing frequency).

The in-phase permittivity versus electrical conductivity at different excitation frequencies is shown in Figure 4. Permittivity is inversely proportional to frequency, with the highest value of permittivity occurring at low frequencies and gradually falling with increased frequencies. The conductivity, on the other hand, is proportional to the frequency, lowest at the low frequencies and rising with an increasing frequency. A similar frequency dependence has also been observed in CB-reinforced styrene butadiene rubber [22]. As seen in Figure 4, the permittivity and conductivity of the samples significantly change after they are compressed and thermally aged. Contrary to the results from the eddy-current analysis, both the conductivity and permittivity decrease when the samples are thermally aged. Lower conductivity values after thermal aging of the rubber material containing carbon black have previously been observed [6]. This was explained by the debonding of polymer chains from the carbon black particle surfaces or the scission of polymer chains by means of oxidization or irradiation. This means changes in the interactions between the rubber matrix, CB particles, and graphene flakes have resulted in changes in conductivity.

The filler material, which may include CB and/or the GP25, creates a conductive network in the polymer matrix, allowing for current conduction. When the conductive filler concentration exceeds the percolation threshold of the polymer matrix, charges can flow through the conductive network formed by these conductive fillers. Alternatively, when the gap between the conductive particles is sufficiently small, charges can jump between the localized sites within the polymer matrix, hopping from one conductive particle to another [23]. The direction of the AC electric field in the electrical impedance measurement is along the longitudinal axis of the cylindrical sample. However, the eddy currents created by the applied AC magnetic excitation field are circles with different radial distances from the origin. The contrary results from the eddy-current and impedance measurements indicate that the conductive CB and GP25 fillers are no longer randomly distributed in the sample and that there is anisotropic electrical percolation in the rubber material. The directional dependency could have been caused by inducing directional shear flow during the mixing of the pre-vulcanized materials or by subjecting the samples to compression, where the compression direction was along the longitudinal axis. Electrical conductivity anisotropy in rubber materials was observed earlier [24,25] when rubber samples were subjected to mechanical loading. 

The permittivity and conductivity of the 0.5 wt% GP25 sample differ significantly from the samples with other GP25 concentrations and have higher values (as was also discovered for the other analysis methods presented). Compared to the eddy-current responses, the permittivity and conductivity variations caused by the aging process are greater in the electrical impedance measurements. The conductive network changes due to compression and the aging process, and the conductivity in the impedance measurements decrease due to thermal aging, meaning that the conductive network along the longitudinal axis of the samples deteriorates. However, the in-phase eddy-current signal, which is related to the conductivity of the material, increases after thermal aging. This suggests an increase in the conductivity network in the polar plane around the center of the samples. 

### 3.3. Hardness Measurements

**Figure 5:**
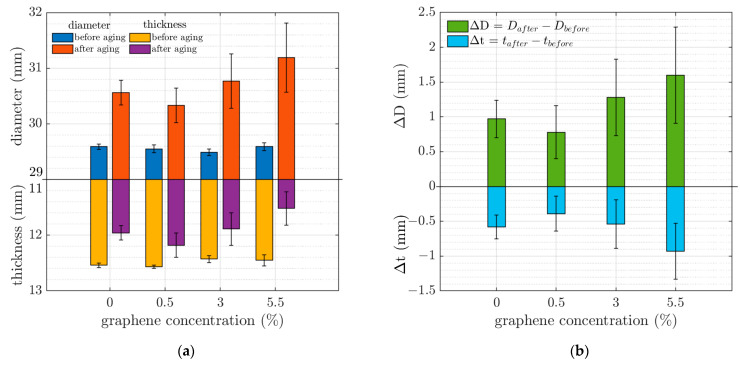
(**a**) Diameter and thickness of samples before and after aging and (**b**) variation in diameter and thickness of samples due to the aging process.

Figure 5a shows the dimensions of the samples with a range of GP25 concentrations before and after aging. The aging process has caused the rubber samples to compress, subsequently increasing their diameter. Figure 5b shows the changes in the diameter and thickness of the samples with various GP25 concentrations after the aging process. It is evident that the sample with 0.5 wt% GP25 has experienced the least deformation from compression compared to the samples with 0, 3, and 5.5 wt% graphene. This could be due to a better graphene dispersion in the 0.5 wt% samples or aggregation of graphene in samples with 3 wt% and 5.5 wt% GP25 graphene filler. However, the morphology of the samples should be further investigated to confirm the presence of graphene aggregations or the degree of uniformity of the graphene dispersion. 

**Figure 6:**
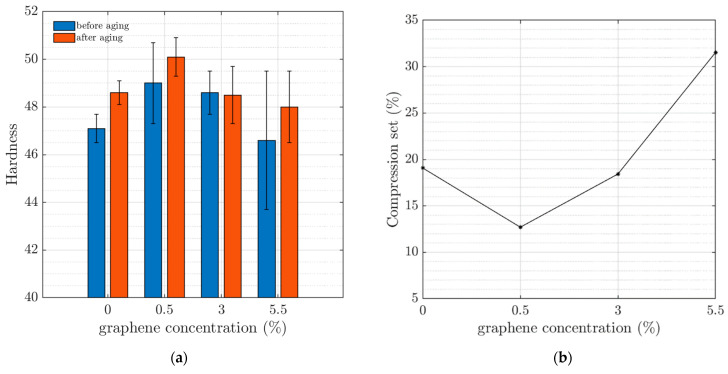
(**a**) Average and standard deviation (error bars) of the hardness measurements of the samples at different graphene concentrations before/after aging. (**b**) The compression set of samples as a function of the graphene concentration.

The results of the hardness measurements are shown in Figure 6a. In general, there was no large difference in the hardness of the samples observed with the addition of GP25 before or after aging. However, an increase in the hardness of the sample with 0.5 wt% GP25 can be observed. There were larger variations in the hardness measurements (larger standard deviation) in the 5.5 wt% graphene sample, which could be due to the dispersion of the GP25; however, this has not been verified. The compression set was also calculated for the samples, and the results are shown in Figure 6b. The sample with 0.5 wt% graphene has the lowest compression set and the best recovery back to the original dimensions after compression compared to the other samples containing GP25. 

## 4. Conclusions

Regardless of the resistivity level of the samples, an eddy-current response could be obtained for all rubber samples. There was an increasing eddy-current response of the samples with up to 30 wt% CB. Further, with increasing amounts of CB, there was no large increase in signal, meaning that the percolation level was reached at 30 wt% CB. There was a small change in the eddy-current response when the CB was replaced with increasing levels of GP25 for the non-aged samples; however, some differences were observed in the samples that had undergone the accelerated aging process. 

The substitution of 0.5 wt% GP25 for CB in the 30 wt% CB formulation gave a slight decrease in permanent mechanical deformation after 30 days of accelerated aging; however, the 3 wt% did not provide a difference, and the 5.5 wt% graphene had an increased permanent deformation compared to the sample with no graphene substitution. This could be a result of the percolation level already being reached or the destruction or agglomeration of the large graphene flakes during processing, resulting in little change compared to the samples containing only CB. Hardness was not significantly changed in materials containing GP25, yet a slight increase could be noticed in the 0.5 wt% GP25 sample. The same sample had the lowest compression set, meaning it had the highest ability to recover to its original shape and dimensions compared to the other concentrations of graphene. 

For the electrical impedance measurements, a very large difference between the non-aged and aged samples was observed. Also, the graphene concentration caused variation in the values of both permittivity and conductivity. The eddy-current response of the samples increased when thermally aged, indicating an increase in sample electrical conductivity or dielectric response. This is contrary to the electrical impedance measurements that showed reductions in both conductivity and dielectric response of the thermally aged samples. This difference could be attributed to the fact that the probing electric field direction is different with respect to the mechanical load during thermal aging when using the two analysis techniques. This suggests that thermal aging, in combination with applied mechanical load, can create an anisotropy in conductivity or dielectric response in the samples.

The results obtained from the eddy-current method demonstrate the feasibility of assessing the aging of EPDM rubber containing CB. This presents a non-destructive method requiring no contact to evaluate the state of the EPDM rubber materials, including sealants, membranes, gaskets, and more. The technique offers several advantages, such as its rapid measurement capabilities and the absence of complex or costly equipment requirements, making it accessible and cost-effective for routine inspections or quality control purposes. This enables real-time monitoring for predictive maintenance, leading to cost reductions, prevention of failures, and enhanced safety, particularly in harsh and challenging environments.

## Figures and Tables

**Table 1:** EPDM rubber composite for all the samples.

CB (wt%)	10.0	15.0	20.0	25.0	30.0	35.0	37.0	40.0	-	-	-	-
GP25 (wt%)	-	-	-	-	-	-	-	-	0	0.5	3.0	5.5
EPDM Polymer 1 (phr *)	100	100	100	100	100	100	100	100	100	100	100	100
Carbon Black N 550 (phr)	12	20	31.2	46.7	70	109	133	133	70	68.8	63	57.1
2Dx GP25 (phr)	0	0	0	0	0	0	0	0	0	1.2	7	12.9
Paraffinic oil (phr)	0	5	16.2	31.7	55	94	118	91	55	55	55	55
Stearic acid (phr)	1	1	1	1	1	1	1	1	1	1	1	1
Activator EDMA (phr)	1.5	1.5	1.5	1.5	1.5	1.5	1.5	1.5	1.5	1.5	1.5	1.5
Peroxide Di(tert-butylperoxyisopropyl) benzene (phr)	6	6	6	6	6	6	6	6	6	6	6	6

* phr—parts per hundred rubbers.

## Data Availability

The data presented in this study are available on request from the corresponding author.

## References

[1] Syao O., Malysheva G.V. (2014). Properties and Application of Rubber-Based Sealants. Polym. Sci. Ser. D.

[2] South J.T., Case S.W., Reifsnider K.L. (2003). Effects of Thermal Aging on The Mechanical Properties of Natural Rubber. Rubber Chem. Technol..

[3] Rahman M.M., Khan F., Kaiser M.S., Ahmed S.R. (2018). Effect of Thermal Ageing on Mechanical Behavior of Synthetic and Natural Rubber Dominated Short Flat Bars. AIP Conf. Proc..

[4] Choi J.-H., Kang H.J., Jeong H.-Y., Lee T.-S., Yoon S.-J. (2005). Heat Aging Effects on the Material Property and the Fatigue Life of Vulcanized Natural Rubber, and Fatigue Life Prediction Equations. J. Mech. Sci. Technol..

[5] Eatah A.I., Ghani A.A., Hashem A.A. (1989). Thermal Ageing Dependence of Electrical Conductivity for Butyl Rubber (IIR) Loaded with HAF Carbon Black. Polym. Degrad. Stab..

[6] Schwartz G.A., Cerveny S., Marzocca Á.J., Gerspacher M., Nikiel L. (2003). Thermal Aging of Carbon Black Filled Rubber Compounds. I. Experimental Evidence for Bridging Flocculation. Polymer.

[7] Abdel-Bary E.M., Amin M., Hassan H.H. (1977). Factors Affecting Electrical Conductivity of Carbon Black-Loaded Rubber. I. Effect of Milling Conditions and Thermal-Oxidative Aging on Electrical Conductivity of Haf Carbon Black-Loaded Styrene–Butadiene Rubber. J. Polym. Sci. Polym. Chem. Ed..

[8] Yan X., Guo J., Jiang X. (2022). The Microwave-Absorption Properties and Mechanism of Phenyl Silicone Rubber/CIPs/Graphene Composites after Thermal-Aging in an Elevated Temperature. Sci. Rep..

[9] Mattson B., Stenberg B. (1992). Electrical Conductivity of Thermo-Oxidatively-Degraded EPDM Rubber. Rubber Chem. Technol..

[10] García-Martín J., Gómez-Gil J., Vázquez-Sánchez E. (2011). Non-Destructive Techniques Based on Eddy Current Testing. Sensors.

[11] Mizukami K., Mizutani Y., Todoroki A., Suzuki Y. (2015). Design of Eddy Current-Based Dielectric Constant Meter for Defect Detection in Glass Fiber Reinforced Plastics. NDT Ampmathsemicolon Int..

[12] Jash P., Parashar R.K., Fontanesi C., Mondal P.C. (2021). The Importance of Electrical Impedance Spectroscopy and Equivalent Circuit Analysis on Nanoscale Molecular Electronic Devices. Adv. Funct. Mater..

[13] Potts J.R., Dreyer D.R., Bielawski C.W., Ruoff R.S. (2011). Graphene-Based Polymer Nanocomposites. Polymer.

[14] Kim H., Abdala A.A., Macosko C.W. (2010). Graphene/Polymer Nanocomposites. Macromolecules.

[15] Govindaraj P., Sokolova A., Salim N., Juodkazis S., Fuss F.K., Fox B., Hameed N. (2021). Distribution States of Graphene in Polymer Nanocomposites: A Review. Compos. Part B Eng..

[16] Zhang H., Xing W., Li H., Xie Z., Huang G., Wu J. (2019). Fundamental Researches on Graphene/Rubber Nanocomposites. Adv. Ind. Eng. Polym. Res..

[17] (2019). Rubber, Vulcanized or Thermoplastic—Determination of Compression Set—Part 1: At Ambient or Elevated Temperatures.

[18] (2018). Standard Test Methods for Rubber Property Compression Set.

[19] (2003). Plastics and Ebonite—Determination of Indentation Hardness by Means of a Durometer (Shore Hardness).

[20] (2021). Standard Test Method for Rubber Property—Durometer Hardness.

[21] Bartosik D., Szadkowski B., Kuśmierek M., Rybiński P., Mirkhodzhaev U., Marzec A. (2022). Advanced Ethylene-Propylene-Diene (EPDM) Rubber Composites Filled with Raw Silicon Carbide or Hybrid Systems with Different Conventional Fillers. Polymers.

[22] Fritzsche J., Klüppel M. (2011). Structural Dynamics and Interfacial Properties of Filler-Reinforced Elastomers. J. Phys. Condens. Matter.

[23] Mohanraj G.T., Chaki T.K., Chakraborty A., Khastgir D. (2007). Measurement of AC Conductivity and Dielectric Properties of Flexible Conductive Styrene–Butadiene Rubber-Carbon Black Composites. J. Appl. Polym. Sci..

[24] Mallik H., Gupta N., Sarkar A. (2002). Anisotropic Electrical Conduction in Gum Arabica—A Biopolymer. Mater. Sci. Eng. C.

[25] Kueseng P., Sae-oui P., Sirisinha C., Jacob K.I., Rattanasom N. (2013). Anisotropic Studies of Multi-Wall Carbon Nanotube (MWCNT)-Filled Natural Rubber (NR) and Nitrile Rubber (NBR) Blends. Polym. Test..

